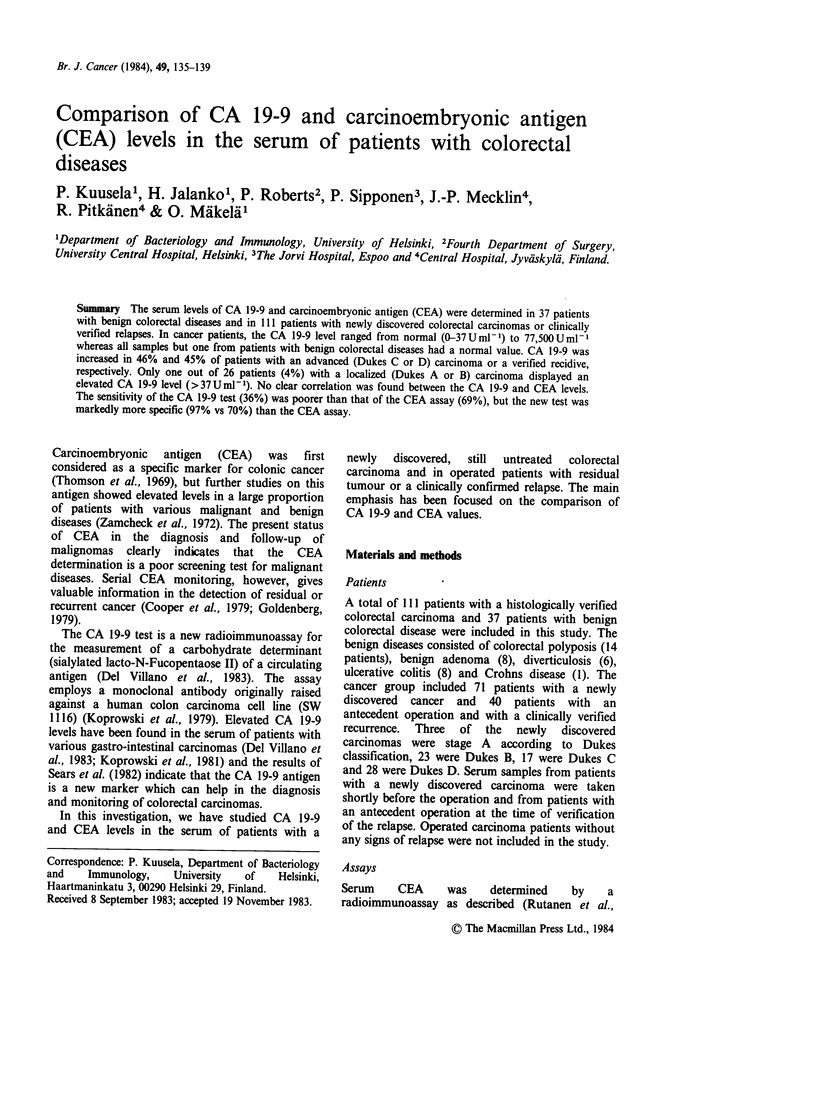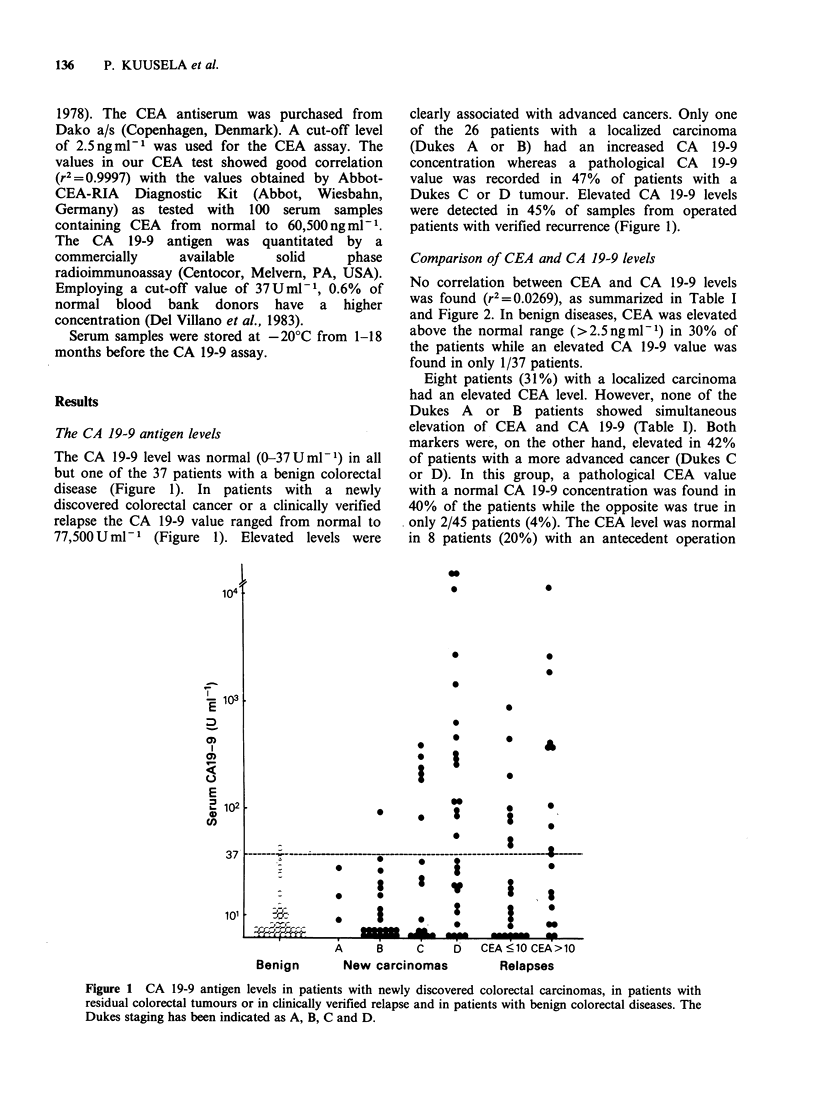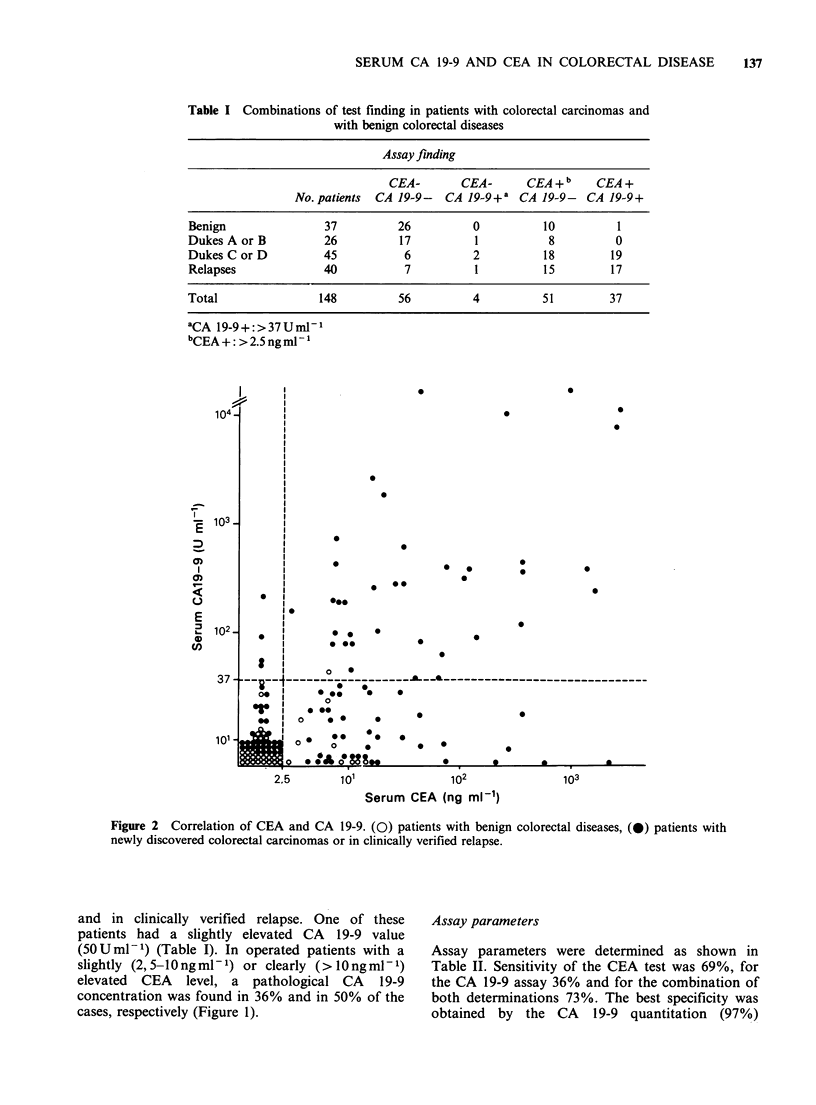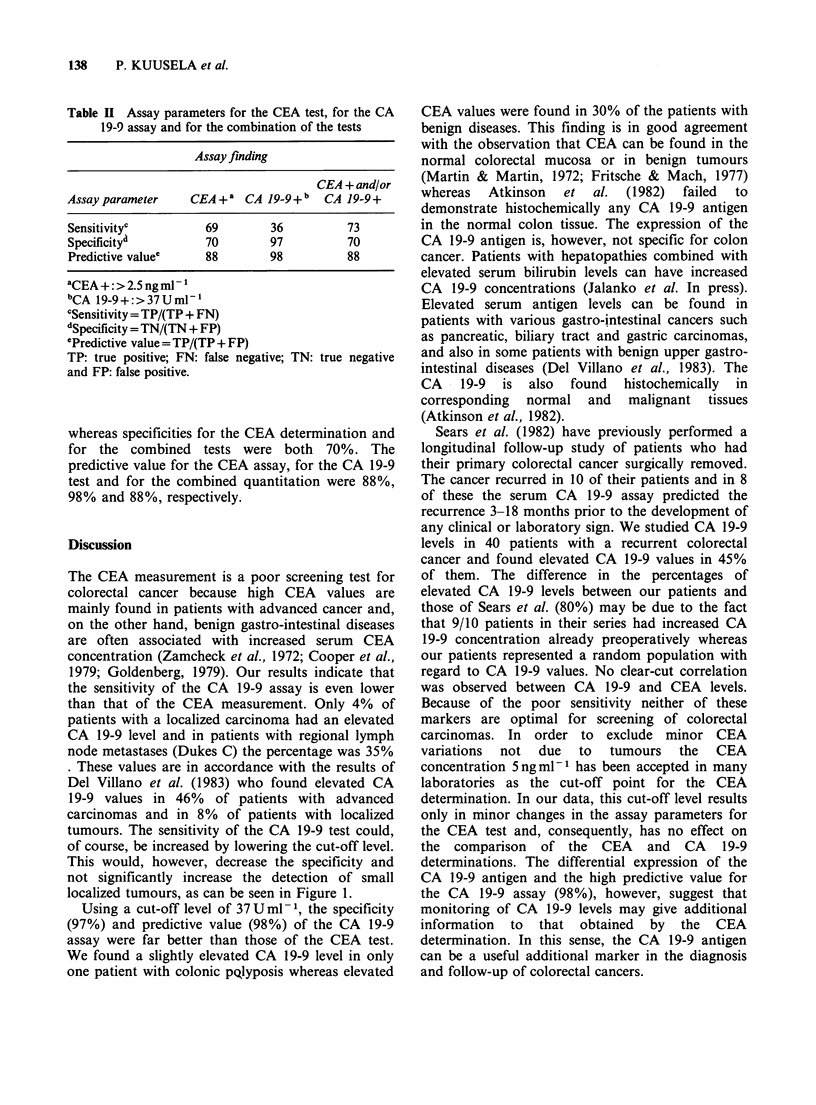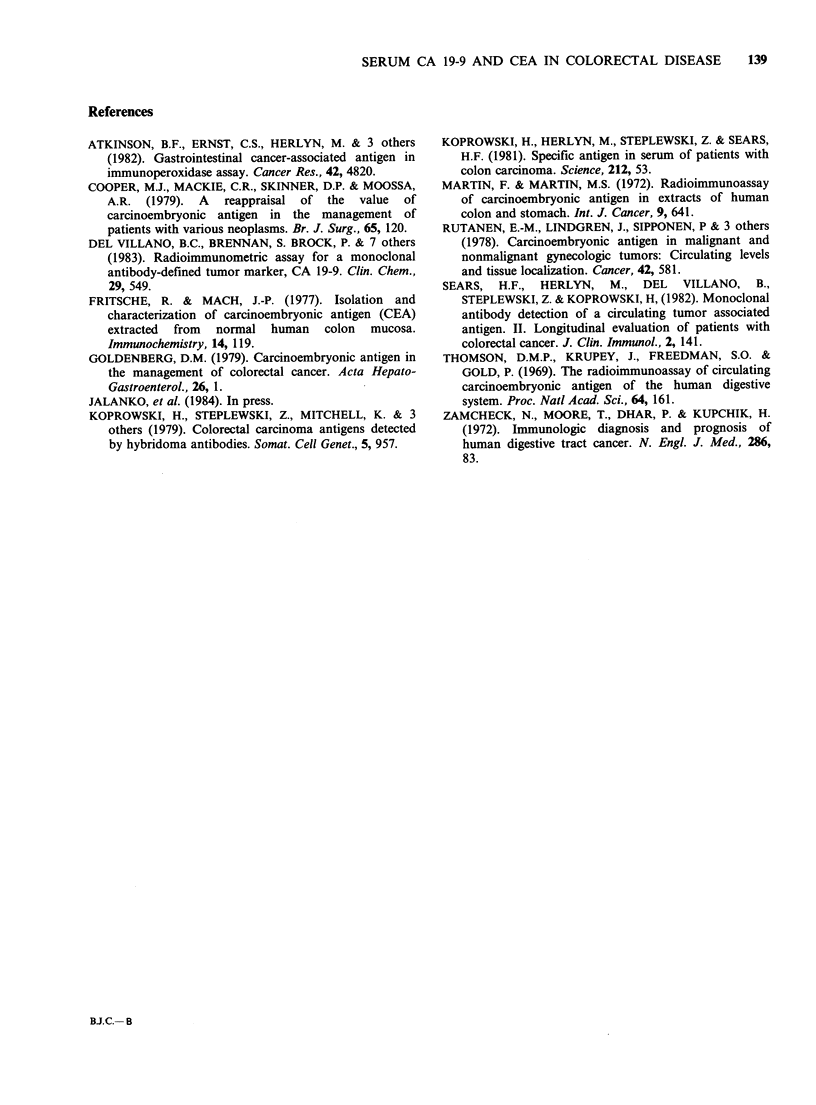# Comparison of CA 19-9 and carcinoembryonic antigen (CEA) levels in the serum of patients with colorectal diseases.

**DOI:** 10.1038/bjc.1984.25

**Published:** 1984-02

**Authors:** P. Kuusela, H. Jalanko, P. Roberts, P. Sipponen, J. P. Mecklin, R. Pitkänen, O. Mäkelä

## Abstract

The serum levels of CA 19-9 and carcinoembryonic antigen (CEA) were determined in 37 patients with benign colorectal diseases and in 111 patients with newly discovered colorectal carcinomas or clinically verified relapses. In cancer patients, the CA 19-9 level ranged from normal (0-37 U ml-1) to 77,500 U ml-1 whereas all samples but one from patients with benign colorectal diseases had a normal value. CA 19-9 was increased in 46% and 45% of patients with an advanced (Dukes C or D) carcinoma or a verified recidive, respectively. Only one out of 26 patients (4%) with a localized (Dukes A or B) carcinoma displayed an elevated CA 19-9 level (greater than 37 U ml-1). No clear correlation was found between the CA 19-9 and CEA levels. The sensitivity of the CA 19-9 test (36%) was poorer than that of the CEA assay (69%), but the new test was markedly more specific (97% vs 70%) than the CEA assay.


*Br. J. Cancer* (1984), **49**, 135–139

### Comparison of CA 19-9 and carcinoembryonic antigen (CEA) levels in the serum of patients with colorectal diseases

P. Kuusela[1], H. Jalanko[1], P. Roberts[2], P. Sipponen[3], J.-P. Mecklin[4], R. Pitkänen[4] & O. Mäkelä[1]

[1]*Department of Bacteriology and Immunology, University of Helsinki,* [2]*Fourth Department of Surgery, University Central Hospital, Helsinki,* [3]*The Jorvi Hospital, Espoo and* [4]*Central Hospital, Jyväskylä, Finland.*

**Summary** The serum levels of CA 19-9 and carcinoembryonic antigen (CEA) were determined in 37 patients with benign colorectal diseases and in 111 patients with newly discovered colorectal carcinomas or clinically verified relapses. In cancer patients, the CA 19-9 level ranged from normal (0–37 U ml$^{-1}$) to 77,500 U ml$^{-1}$ whereas all samples but one from patients with benign colorectal diseases had a normal value. CA 19-9 was increased in 46% and 45% of patients with an advanced (Dukes C or D) carcinoma or a verified recidive, respectively. Only one out of 26 patients (4%) with a localized (Dukes A or B) carcinoma displayed an elevated CA 19-9 level (>37 U ml$^{-1}$). No clear correlation was found between the CA 19-9 and CEA levels. The sensitivity of the CA 19-9 test (36%) was poorer than that of the CEA assay (69%), but the new test was markedly more specific (97% vs 70%) than the CEA assay.

Carcinoembryonic antigen (CEA) was first considered as a specific marker for colonic cancer (Thomson *et al.*, 1969), but further studies on this antigen showed elevated levels in a large proportion of patients with various malignant and benign diseases (Zamcheck *et al.*, 1972). The present status of CEA in the diagnosis and follow-up of malignomas clearly indicates that the CEA determination is a poor screening test for malignant diseases. Serial CEA monitoring, however, gives valuable information in the detection of residual or recurrent cancer (Cooper *et al.*, 1979; Goldenberg, 1979).

The CA 19-9 test is a new radioimmunoassay for the measurement of a carbohydrate determinant (sialylated lacto-N-Fucopentaose II) of a circulating antigen (Del Villano *et al.*, 1983). The assay employs a monoclonal antibody originally raised against a human colon carcinoma cell line (SW 1116) (Koprowski *et al.*, 1979). Elevated CA 19-9 levels have been found in the serum of patients with various gastro-intestinal carcinomas (Del Villano *et al.*, 1983; Koprowski *et al.*, 1981) and the results of Sears *et al.* (1982) indicate that the CA 19-9 antigen is a new marker which can help in the diagnosis and monitoring of colorectal carcinomas.

In this investigation, we have studied CA 19-9 and CEA levels in the serum of patients with a newly discovered, still untreated colorectal carcinoma and in operated patients with residual tumour or a clinically confirmed relapse. The main emphasis has been focused on the comparison of CA 19-9 and CEA values.

Correspondence: P. Kuusela, Department of Bacteriology and Immunology, University of Helsinki, Haartmaninkatu 3, 00290 Helsinki 29, Finland.
Received 8 September 1983; accepted 19 November 1983.

### Materials and methods

### *Patients*

A total of 111 patients with a histologically verified colorectal carcinoma and 37 patients with benign colorectal disease were included in this study. The benign diseases consisted of colorectal polyposis (14 patients), benign adenoma (8), diverticulosis (6), ulcerative colitis (8) and Crohns disease (1). The cancer group included 71 patients with a newly discovered cancer and 40 patients with an antecedent operation and with a clinically verified recurrence. Three of the newly discovered carcinomas were stage A according to Dukes classification, 23 were Dukes B, 17 were Dukes C and 28 were Dukes D. Serum samples from patients with a newly discovered carcinoma were taken shortly before the operation and from patients with an antecedent operation at the time of verification of the relapse. Operated carcinoma patients without any signs of relapse were not included in the study.

### *Assays*

Serum CEA was determined by a radioimmunoassay as described (Rutanen *et al.*,

© The Macmillan Press Ltd., 1984

1978). The CEA antiserum was purchased from Dako a/s (Copenhagen, Denmark). A cut-off level of 2.5 ng ml$^{-1}$ was used for the CEA assay. The values in our CEA test showed good correlation ($r^2 = 0.9997$) with the values obtained by Abbot-CEA-RIA Diagnostic Kit (Abbot, Wiesbahn, Germany) as tested with 100 serum samples containing CEA from normal to 60,500 ng ml$^{-1}$. The CA 19-9 antigen was quantitated by a commercially available solid phase radioimmunoassay (Centocor, Melvern, PA, USA). Employing a cut-off value of 37 U ml$^{-1}$, 0.6% of normal blood bank donors have a higher concentration (Del Villano *et al.*, 1983).

Serum samples were stored at −20°C from 1–18 months before the CA 19-9 assay.

### Results

### *The CA 19-9 antigen levels*

The CA 19-9 level was normal (0–37 U ml$^{-1}$) in all but one of the 37 patients with a benign colorectal disease (Figure 1). In patients with a newly discovered colorectal cancer or a clinically verified relapse the CA 19-9 value ranged from normal to 77,500 U ml$^{-1}$ (Figure 1). Elevated levels were clearly associated with advanced cancers. Only one of the 26 patients with a localized carcinoma (Dukes A or B) had an increased CA 19-9 concentration whereas a pathological CA 19-9 value was recorded in 47% of patients with a Dukes C or D tumour. Elevated CA 19-9 levels were detected in 45% of samples from operated patients with verified recurrence (Figure 1).

### *Comparison of CEA and CA 19-9 levels*

No correlation between CEA and CA 19-9 levels was found ($r^2 = 0.0269$), as summarized in Table I and Figure 2. In benign diseases, CEA was elevated above the normal range (>2.5 ng ml$^{-1}$) in 30% of the patients while an elevated CA 19-9 value was found in only 1/37 patients.

Eight patients (31%) with a localized carcinoma had an elevated CEA level. However, none of the Dukes A or B patients showed simultaneous elevation of CEA and CA 19-9 (Table I). Both markers were, on the other hand, elevated in 42% of patients with a more advanced cancer (Dukes C or D). In this group, a pathological CEA value with a normal CA 19-9 concentration was found in 40% of the patients while the opposite was true in only 2/45 patients (4%). The CEA level was normal in 8 patients (20%) with an antecedent operation

**Figure 1** CA 19-9 antigen levels in patients with newly discovered colorectal carcinomas, in patients with residual colorectal tumours or in clinically verified relapse and in patients with benign colorectal diseases. The Dukes staging has been indicated as A, B, C and D.

**Table I** Combinations of test finding in patients with colorectal carcinomas and with benign colorectal diseases

| | | *Assay finding* | | | |
|---|---|---|---|---|---|
| | *No. patients* | *CEA- CA 19-9−* | *CEA- CA 19-9+*[a] | *CEA+*[b] *CA 19-9−* | *CEA+ CA 19-9+* |
| Benign | 37 | 26 | 0 | 10 | 1 |
| Dukes A or B | 26 | 17 | 1 | 8 | 0 |
| Dukes C or D | 45 | 6 | 2 | 18 | 19 |
| Relapses | 40 | 7 | 1 | 15 | 17 |
| Total | 148 | 56 | 4 | 51 | 37 |

[a]CA 19-9+: >37 U ml$^{-1}$

[b]CEA+: >2.5 ng ml$^{-1}$

**Figure 2** Correlation of CEA and CA 19-9. (○) patients with benign colorectal diseases, (●) patients with newly discovered colorectal carcinomas or in clinically verified relapse.

and in clinically verified relapse. One of these patients had a slightly elevated CA 19-9 value (50 U ml$^{-1}$) (Table I). In operated patients with a slightly (2,5–10 ng ml$^{-1}$) or clearly (>10 ng ml$^{-1}$) elevated CEA level, a pathological CA 19-9 concentration was found in 36% and in 50% of the cases, respectively (Figure 1).

*Assay parameters*

Assay parameters were determined as shown in Table II. Sensitivity of the CEA test was 69%, for the CA 19-9 assay 36% and for the combination of both determinations 73%. The best specificity was obtained by the CA 19-9 quantitation (97%)

**Table II** Assay parameters for the CEA test, for the CA 19-9 assay and for the combination of the tests

| | *Assay finding* | | |
|---|---|---|---|
| *Assay parameter* | *CEA+*[a] | *CA 19-9+*[b] | *CEA+and/or CA 19-9+* |
| Sensitivity[c] | 69 | 36 | 73 |
| Specificity[d] | 70 | 97 | 70 |
| Predictive value[e] | 88 | 98 | 88 |

[a]CEA+: >2.5 ng ml$^{-1}$
[b]CA 19-9+: >37 U ml$^{-1}$
[c]Sensitivity = TP/(TP + FN)
[d]Specificity = TN/(TN + FP)
[e]Predictive value = TP/(TP + FP)
TP: true positive; FN: false negative; TN: true negative and FP: false positive.

whereas specificities for the CEA determination and for the combined tests were both 70%. The predictive value for the CEA assay, for the CA 19-9 test and for the combined quantitation were 88%, 98% and 88%, respectively.

### Discussion

The CEA measurement is a poor screening test for colorectal cancer because high CEA values are mainly found in patients with advanced cancer and, on the other hand, benign gastro-intestinal diseases are often associated with increased serum CEA concentration (Zamcheck *et al.*, 1972; Cooper *et al.*, 1979; Goldenberg, 1979). Our results indicate that the sensitivity of the CA 19-9 assay is even lower than that of the CEA measurement. Only 4% of patients with a localized carcinoma had an elevated CA 19-9 level and in patients with regional lymph node metastases (Dukes C) the percentage was 35% . These values are in accordance with the results of Del Villano *et al.* (1983) who found elevated CA 19-9 values in 46% of patients with advanced carcinomas and in 8% of patients with localized tumours. The sensitivity of the CA 19-9 test could, of course, be increased by lowering the cut-off level. This would, however, decrease the specificity and not significantly increase the detection of small localized tumours, as can be seen in Figure 1.

Using a cut-off level of 37 U ml$^{-1}$, the specificity (97%) and predictive value (98%) of the CA 19-9 assay were far better than those of the CEA test. We found a slightly elevated CA 19-9 level in only one patient with colonic polyposis whereas elevated CEA values were found in 30% of the patients with benign diseases. This finding is in good agreement with the observation that CEA can be found in the normal colorectal mucosa or in benign tumours (Martin & Martin, 1972; Fritsche & Mach, 1977) whereas Atkinson *et al.* (1982) failed to demonstrate histochemically any CA 19-9 antigen in the normal colon tissue. The expression of the CA 19-9 antigen is, however, not specific for colon cancer. Patients with hepatopathies combined with elevated serum bilirubin levels can have increased CA 19-9 concentrations (Jalanko *et al.* In press). Elevated serum antigen levels can be found in patients with various gastro-intestinal cancers such as pancreatic, biliary tract and gastric carcinomas, and also in some patients with benign upper gastro-intestinal diseases (Del Villano *et al.*, 1983). The CA 19-9 is also found histochemically in corresponding normal and malignant tissues (Atkinson *et al.*, 1982).

Sears *et al.* (1982) have previously performed a longitudinal follow-up study of patients who had their primary colorectal cancer surgically removed. The cancer recurred in 10 of their patients and in 8 of these the serum CA 19-9 assay predicted the recurrence 3–18 months prior to the development of any clinical or laboratory sign. We studied CA 19-9 levels in 40 patients with a recurrent colorectal cancer and found elevated CA 19-9 values in 45% of them. The difference in the percentages of elevated CA 19-9 levels between our patients and those of Sears *et al.* (80%) may be due to the fact that 9/10 patients in their series had increased CA 19-9 concentration already preoperatively whereas our patients represented a random population with regard to CA 19-9 values. No clear-cut correlation was observed between CA 19-9 and CEA levels. Because of the poor sensitivity neither of these markers are optimal for screening of colorectal carcinomas. In order to exclude minor CEA variations not due to tumours the CEA concentration 5 ng ml$^{-1}$ has been accepted in many laboratories as the cut-off point for the CEA determination. In our data, this cut-off level results only in minor changes in the assay parameters for the CEA test and, consequently, has no effect on the comparison of the CEA and CA 19-9 determinations. The differential expression of the CA 19-9 antigen and the high predictive value for the CA 19-9 assay (98%), however, suggest that monitoring of CA 19-9 levels may give additional information to that obtained by the CEA determination. In this sense, the CA 19-9 antigen can be a useful additional marker in the diagnosis and follow-up of colorectal cancers.

### References

ATKINSON, B.F., ERNST, C.S., HERLYN, M. & 3 others (1982). Gastrointestinal cancer-associated antigen in immunoperoxidase assay. *Cancer Res.*, **42**, 4820.

COOPER, M.J., MACKIE, C.R., SKINNER, D.P. & MOOSSA, A.R. (1979). A reappraisal of the value of carcinoembryonic antigen in the management of patients with various neoplasms. *Br. J. Surg.*, **65**, 120.

DEL VILLANO, B.C., BRENNAN, S. BROCK, P. & 7 others (1983). Radioimmunometric assay for a monoclonal antibody-defined tumor marker, CA 19-9. *Clin. Chem.*, **29**, 549.

FRITSCHE, R. & MACH, J.-P. (1977). Isolation and characterization of carcinoembryonic antigen (CEA) extracted from normal human colon mucosa. *Immunochemistry*, **14**, 119.

GOLDENBERG, D.M. (1979). Carcinoembryonic antigen in the management of colorectal cancer. *Acta Hepato-Gastroenterol.*, **26**, 1.

JALANKO, *et al.* (1984). In press.

KOPROWSKI, H., STEPLEWSKI, Z., MITCHELL, K. & 3 others (1979). Colorectal carcinoma antigens detected by hybridoma antibodies. *Somat. Cell Genet.*, **5**, 957.

KOPROWSKI, H., HERLYN, M., STEPLEWSKI, Z. & SEARS, H.F. (1981). Specific antigen in serum of patients with colon carcinoma. *Science*, **212**, 53.

MARTIN, F. & MARTIN, M.S. (1972). Radioimmunoassay of carcinoembryonic antigen in extracts of human colon and stomach. *Int. J. Cancer*, **9**, 641.

RUTANEN, E.-M., LINDGREN, J., SIPPONEN, P & 3 others (1978). Carcinoembryonic antigen in malignant and nonmalignant gynecologic tumors: Circulating levels and tissue localization. *Cancer*, **42**, 581.

SEARS, H.F., HERLYN, M., DEL VILLANO, B., STEPLEWSKI, Z. & KOPROWSKI, H, (1982). Monoclonal antibody detection of a circulating tumor associated antigen. II. Longitudinal evaluation of patients with colorectal cancer. *J. Clin. Immunol.*, **2**, 141.

THOMSON, D.M.P., KRUPEY, J., FREEDMAN, S.O. & GOLD, P. (1969). The radioimmunoassay of circulating carcinoembryonic antigen of the human digestive system. *Proc. Natl Acad. Sci.*, **64**, 161.

ZAMCHECK, N., MOORE, T., DHAR, P. & KUPCHIK, H. (1972). Immunologic diagnosis and prognosis of human digestive tract cancer. *N. Engl. J. Med.*, **286**, 83.